# Plasma-Assisted Atomic Layer Deposition of High-Density Ni Nanoparticles for Amorphous In-Ga-Zn-O Thin Film Transistor Memory

**DOI:** 10.1186/s11671-017-1925-z

**Published:** 2017-02-21

**Authors:** Shi-Bing Qian, Yong-Ping Wang, Yan Shao, Wen-Jun Liu, Shi-Jin Ding

**Affiliations:** State Key Laboratory of ASIC and System, School of Microelectronics, Fudan University, 200433 Shanghai, China

**Keywords:** Ni nanoparticles, Plasma-assisted atomic layer deposition, In-Ga-Zn-O, Memory

## Abstract

For the first time, the growth of Ni nanoparticles (NPs) was explored by plasma-assisted atomic layer deposition (ALD) technique using NiCp_2_ and NH_3_ precursors. Influences of substrate temperature and deposition cycles on ALD Ni NPs were studied by field emission scanning electron microscope and X-ray photoelectron spectroscopy. By optimizing the process parameters, high-density and uniform Ni NPs were achieved in the case of 280 °C substrate temperature and 50 deposition cycles, exhibiting a density of ~1.5 × 10^12^ cm^−2^ and a small size of 3~4 nm. Further, the above Ni NPs were used as charge storage medium of amorphous indium-gallium-zinc oxide (a-IGZO) thin film transistor (TFT) memory, demonstrating a high storage capacity for electrons. In particular, the nonvolatile memory exhibited an excellent programming characteristic, e.g., a large threshold voltage shift of 8.03 V was obtained after being programmed at 17 V for 5 ms.

## Background

Nickel (Ni) nanoparticles (NPs) have been intensively explored due to their various potential applications, such as magnetic materials [1–3] and steam reforming catalyst [4, 5]. Moreover, Ni NPs have also been investigated as a charge storage medium for nonvolatile memory devices [6–12], which could be attributed to some potential advantages of Ni NPs, including high density of states around the Fermi level, strong charge confinement, a high work function of 5.2 eV, and low diffusivity. Therefore, the employment of Ni NPs as the charge storage medium can obtain a deep potential well in nonvolatile memory devices by selecting appropriate insulators, hereby ensuring good data retention. To obtain high-density Ni NPs, most researchers performed rapid thermal annealing of ultra-thin Ni films [7–10]. However, this technique usually requires a high annealing temperature (e.g., 600~900 °C), which undoubtedly causes crystallization for most of high dielectric constant insulators, and also exhibits temperature incompatibility with the standard thin film transistor (TFT) process. For example, Tan et al. prepared the Ni NPs with an average size of 15 nm and a relatively low density of ~10^11^ cm^−2^ under the maximum process temperature of 700 °C [6]. In recent years, the technique of atomic layer deposition (ALD), which is based on sequential self-limited and complementary surface chemisorption reactions, has been considered as a perfect thin film deposition method because of its outstanding advantages such as relatively low process temperature, stoichiometric composition, large-area uniformity, precise thickness control, and high conformability [13, 14]. Furthermore, ALD is also a very promising method for preparing high-density metal NPs (e.g., Pt, Ir, and Ru) at relatively low temperature of <350 °C [15–17]. As an example, Liu et al. obtained Ir NPs with a high density of 0.6 × 10^12^ cm^−2^ and an average size of 4.9 nm by ALD at 300 °C for nonvolatile memory applications [17]. Ding et al. also reported that two-dimensional high-density Pt NPs (2 × 10^12^ cm^−2^) were self-assembled on the Al_2_O_3_ film at 300 °C by ALD, which demonstrated noticeable electron trapping capacity in Si-based nonvolatile memory [15].

On the other hand, to develop a fully functional transparent system on a panel, nonvolatile amorphous In-Ga-Zn-O (a-IGZO) TFT memory has been proposed due to easy integration into displays and flexible electronic devices [18–20]. This is attributed to some merits of a-IGZO, such as high electron mobility, good visible-light transparency, excellent uniformity, and low process temperature [21, 22]. Therefore, it is indispensable to explore the electrical information storage ability of a-IGZO TFT memory with innovative material and process. Considering excellent process temperature compatibility between ALD of metal NPs and fabrication of a-IGZO TFT devices, the a-IGZO TFT memory with the charge storage medium of ALD Ni NPs was investigated in this article. Firstly, the ALD growth of Ni NPs on the Al_2_O_3_ film was studied, including influences of substrate temperature and deposition cycles on ALD Ni NPs. Further, by using ALD high-density Ni NPs as charge storage medium, the a-IGZO TFT memory was fabricated and the memory characteristics were measured.

## Methods

Firstly, an around 10-nm Al_2_O_3_ film was deposited on a cleaned p-type silicon wafer by ALD. Subsequently, Ni NPs were grown by plasma-assisted ALD on the surface of the Al_2_O_3_ film using NiCp_2_ and NH_3_ precursors. Herein, the NiCp_2_ precursor was kept at 80 °C; the vapor of NiCp_2_ was pulsed into the reaction chamber with a N_2_ carrier gas. The NH_3_ plasma was generated under a power of 3000 W with a flow rate of 180 sccm. During the ALD process, the working pressure in the deposition chamber was maintained at ~1200 Pa, and the NiCp_2_ and NH_3_ plasma pulse times were fixed at 2 and 20 s, respectively. To obtain the optimal process conditions, the growth of Ni NPs was investigated as a function of substrate temperature and reaction cycles, respectively.

To fabricate the a-IGZO TFT memory device, the cleaned p-type Si (100) wafer with a resistivity of 0.001~0.005 Ω cm was used as the starting substrate serving as the back gate of the device. Then, a 35-nm Al_2_O_3_ film, Ni NPs, and a 7-nm Al_2_O_3_ film were successively deposited by ALD in the same chamber, which were used as the blocking layer, charge storage medium, and tunneling layer of the memory, respectively. After that, a 40-nm a-IGZO film was deposited by radio frequency (RF) magnetron sputtering at room temperature using an InGaZnO_4_ target under the following conditions: Ar = 50 sccm, RF power = 110 W, and working pressure = 0.87 Pa. Subsequently, the active channel was defined by photolithography and wet etching (diluted HCl acid). Source and drain contacts of Ti/Au (30 nm/70 nm) layers were formed by e-beam evaporation and a lift-off technique. Finally, the fabricated device was annealed at 250 °C in O_2_ for 5 min. The control TFT device without Ni NPs was also fabricated for comparison.

The morphologies and compositions of ALD Ni NPs were characterized by field emission scanning electron microscope (FE-SEM) (JSM-6700F, JEOL, Tokyo, Japan) and X-ray photoelectron spectroscopy (XPS) (Kratos Axis Ultra DLD), respectively. The thicknesses of Al_2_O_3_ and IGZO films were measured with an ellipsometer (Sopra GES-SE, France). The electrical measurements were performed on the devices with a channel length (L) of 10 μm and a channel width (W) of 100 μm using a semiconductor device analyzer (Agilent B1500A) at room temperature in a dark box. The threshold voltage (*V*_th_) is defined as the gate voltage at which the drain current equals the W/L × 10^−9^ A when *V*_ds_ is fixed at 0.1 V.

## Results and Discussion

**Fig. 1:**
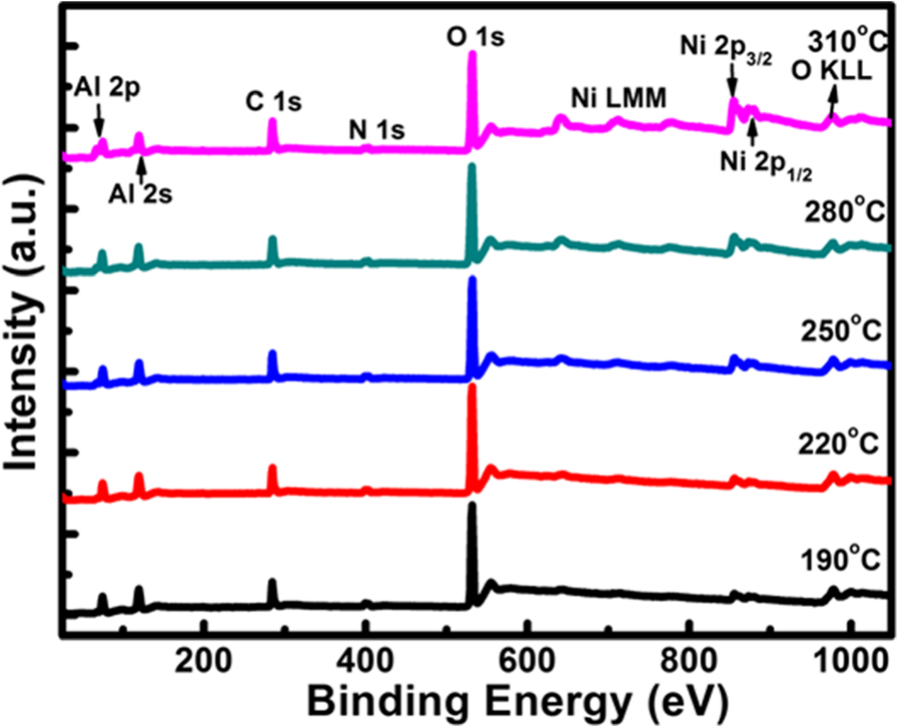
Survey XPS spectra of as-deposited Ni on the Al_2_O_3_ film as a function of substrate temperature for 100 deposition cycles

Figure 1 shows the survey XPS spectra of the as-deposited Ni on the Al_2_O_3_ film at different substrate temperatures in the case of 100 deposition cycles. It is found that the photoelectron peaks of Ni, C, N, Al, and O elements are observed, which should stem from both the deposited Ni and the Al_2_O_3_ backing film. The intensity of the Ni 2p peak increases distinctly with raising the substrate temperature from 190 to 310 °C. This reflects a remarkable increase in the amount of Ni atoms deposited on the Al_2_O_3_ film.

**Fig. 2:**
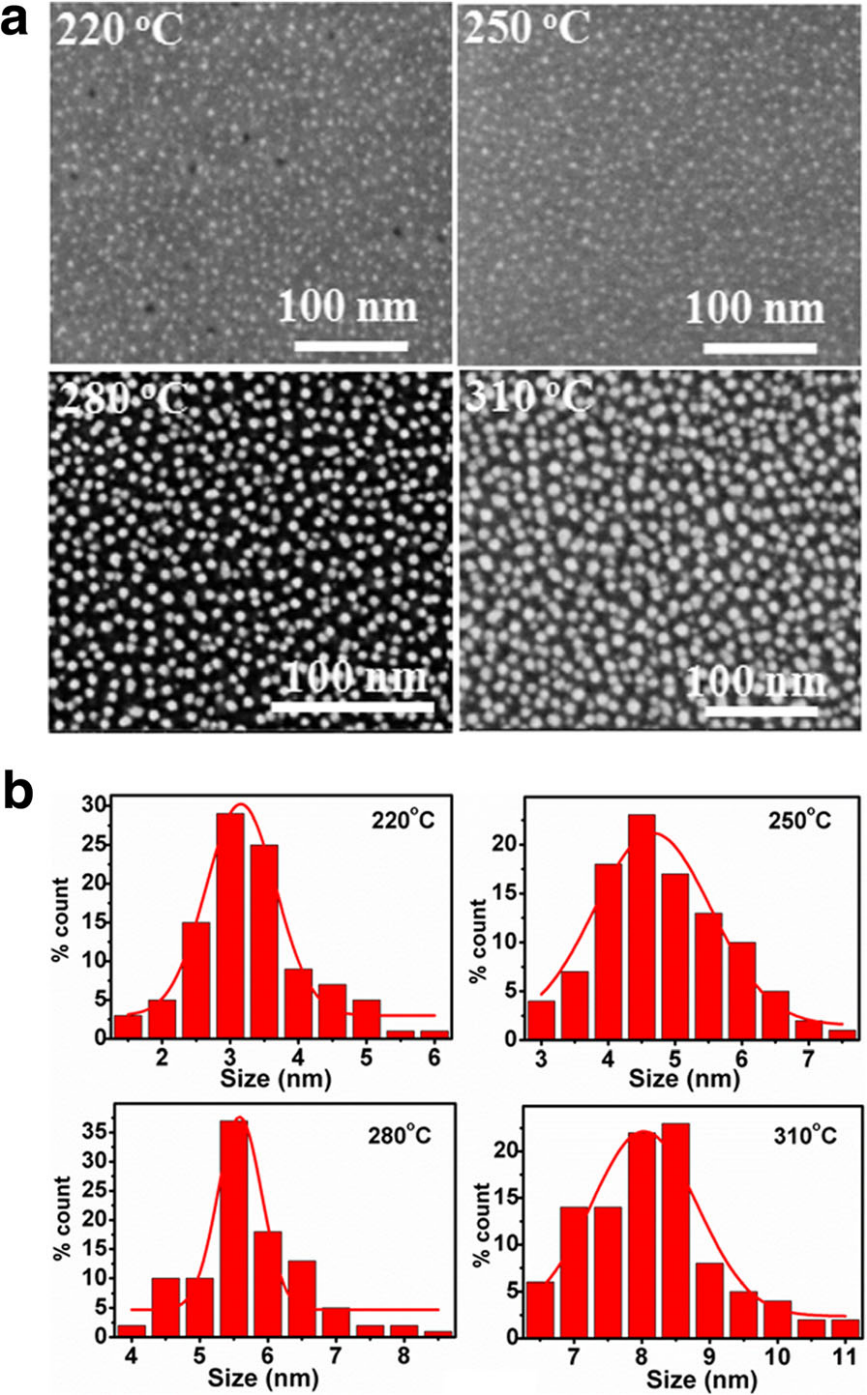
**a** SEM images of ALD Ni NPs on the Al_2_O_3_ film for 100 cycles at different substrate temperatures. **b** The histograms of size distribution of the corresponding Ni NPs, fitted by Gaussian function

In order to observe intuitively the formation of Ni NPs, the surface morphologies of Ni NPs deposited at different substrate temperatures are shown in Fig. 2a. Regarding the substrate temperatures of 220 and 250 ^o^C, the deposited Ni NPs show a small size, nonuniform shapes and a low density of ~5 × 10^11^ cm^−2^. When the substrate temperature increases to 280 °C, the resulting Ni NPs become bigger and denser, demonstrating a density as high as ~1.1 × 10^12^ cm^−2^. As the substrate temperature rises to 310 °C, most of Ni NPs become much bigger and begin to connect with each other, thus exhibiting irregular shapes and a reduced density of ~3 × 10^11^ cm^−2^. This indicates that high-density Ni NPs can be self-assembled on the Al_2_O_3_ film at relatively low temperatures (typically ≤280 °C). Regarding the substrate temperature lower than 250 °C, the relatively slow growth of Ni NPs should be attributed to the lack of adequate activation energy for the chemical reaction [23]. Furthermore, Fig. 2b shows the histograms of size distribution of the corresponding Ni NPs deposited at different substrate temperatures. As the substrate temperature increases from 220 to 310 °C, the mean size of Ni NPs rises from 3.0 to 8.0 nm. Regarding the substrate temperature of 280 °C, the Ni NPs show the narrowest size distribution, which is centered at 5.5 nm.

**Fig. 3:**
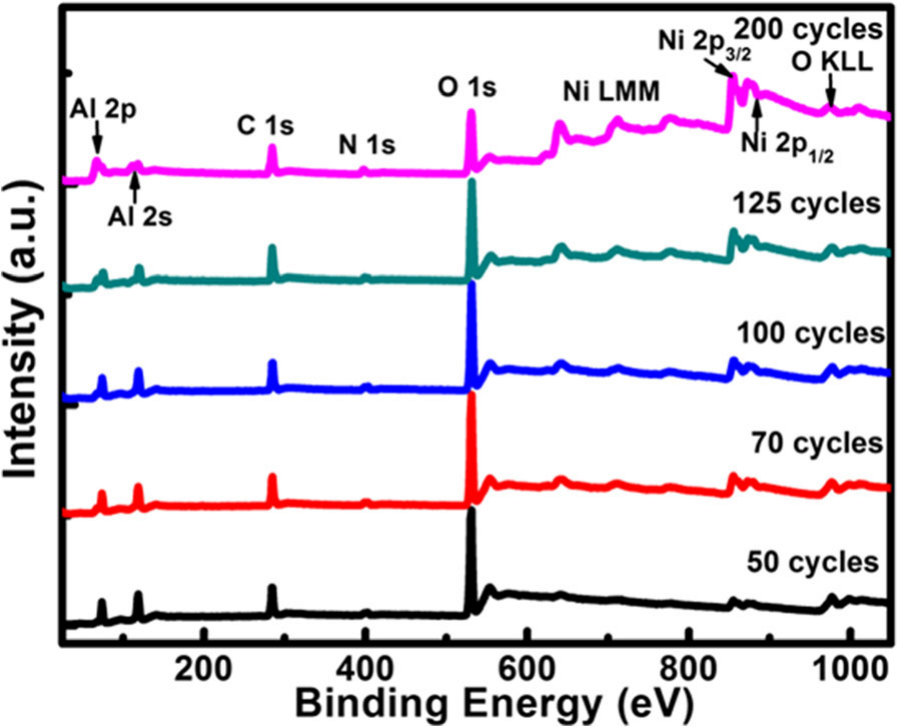
Survey XPS spectra of ALD Ni on the Al_2_O_3_ film as a function of deposition cycles (substrate temperature 280 °C)

With respect to ALD metal NPs, the number of deposition cycles is also a key point. Therefore, the influence of deposition cycles on ALD Ni NPs is investigated while maintaining a constant substrate temperature. Figure 3 shows the survey XPS spectra of as-deposited Ni on the Al_2_O_3_ film as a function of deposition cycles for the substrate temperature of 280 °C. The photoelectron peaks of Ni, C, N, Al, and O elements are still observed. Further, it is found that the intensity ratio of Ni 2p to Al 2p peaks increases gradually with an increment of deposition cycles from 50 to 200; this reflects a marked increase in Ni coverage on the surface of the Al_2_O_3_ film.

**Fig. 4:**
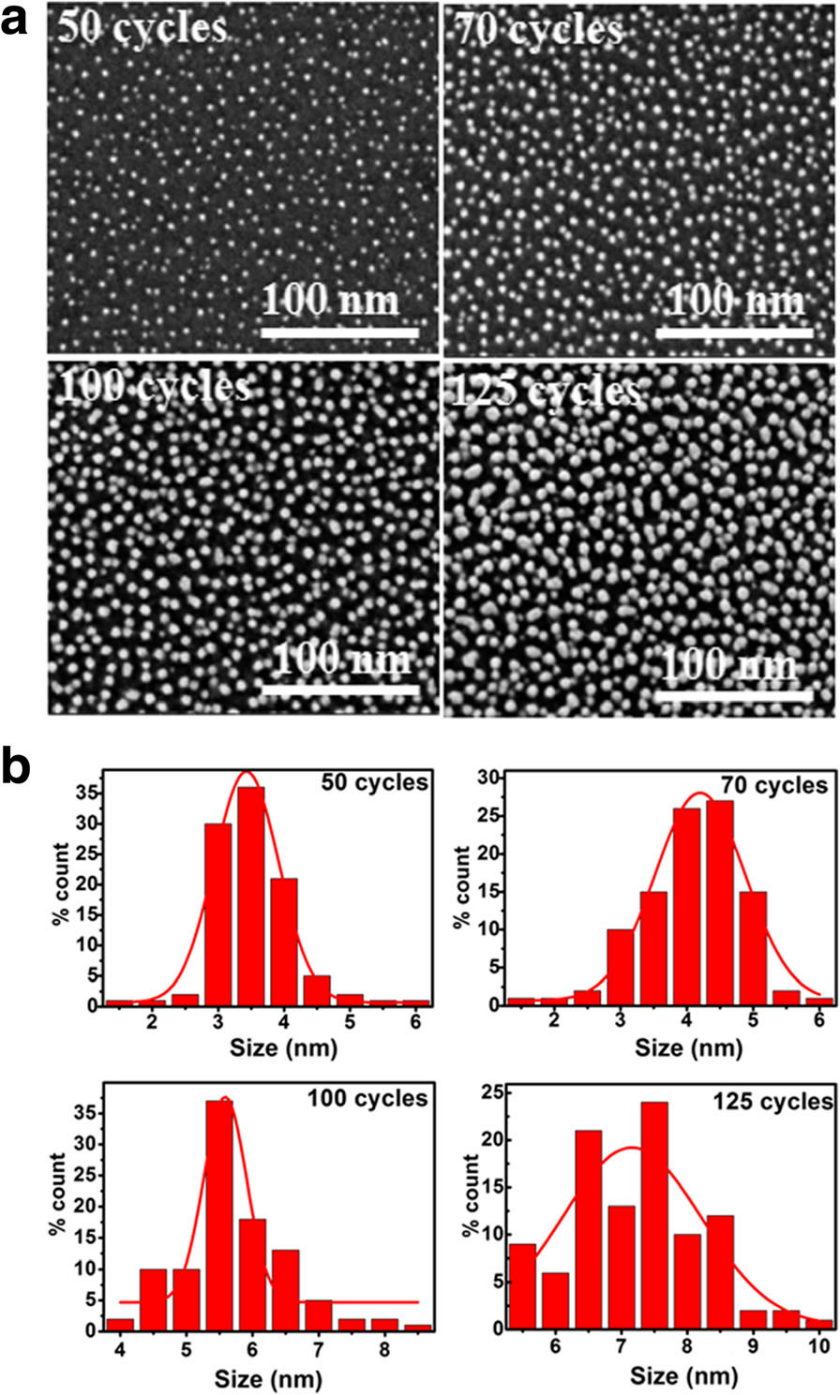
**a** SEM images of ALD Ni NPs on the Al_2_O_3_ film at 280 °C for different deposition cycles. **b** The histograms of size distribution of the corresponding Ni NPs, fitted by Gaussian function

Figure 4a illustrates the surface morphologies of the deposited Ni NPs at 280 °C for different deposition cycles. It is obvious that the Ni NPs become bigger and bigger as the deposition cycles increase from 50 to 125. In the case of ≤70 deposition cycles, the deposited Ni NPs exhibit a density as high as 1.45 × 10^12^~1.56 × 10^12^ cm^−2^ and a small quasi-spherical shape. When the deposition cycles reach 125, the probability of coalescence between neighboring Ni NPs increases remarkably, thus generating the appearance of irregular shapes and a reduced density of 8 × 10^11^ cm^−2^. Further, the histograms of the size distribution of Ni NPs are shown in Fig. 4b. As the deposition cycles increase from 50 to 125, the mean size of Ni NPs rises from 3.5 to 7.0 nm. In terms of 50 deposition cycles, the Ni NPs show the narrowest size distribution. In particular, when the deposition cycles increase to 125, the NPs exhibit a very wide size distribution.

**Fig. 5:**
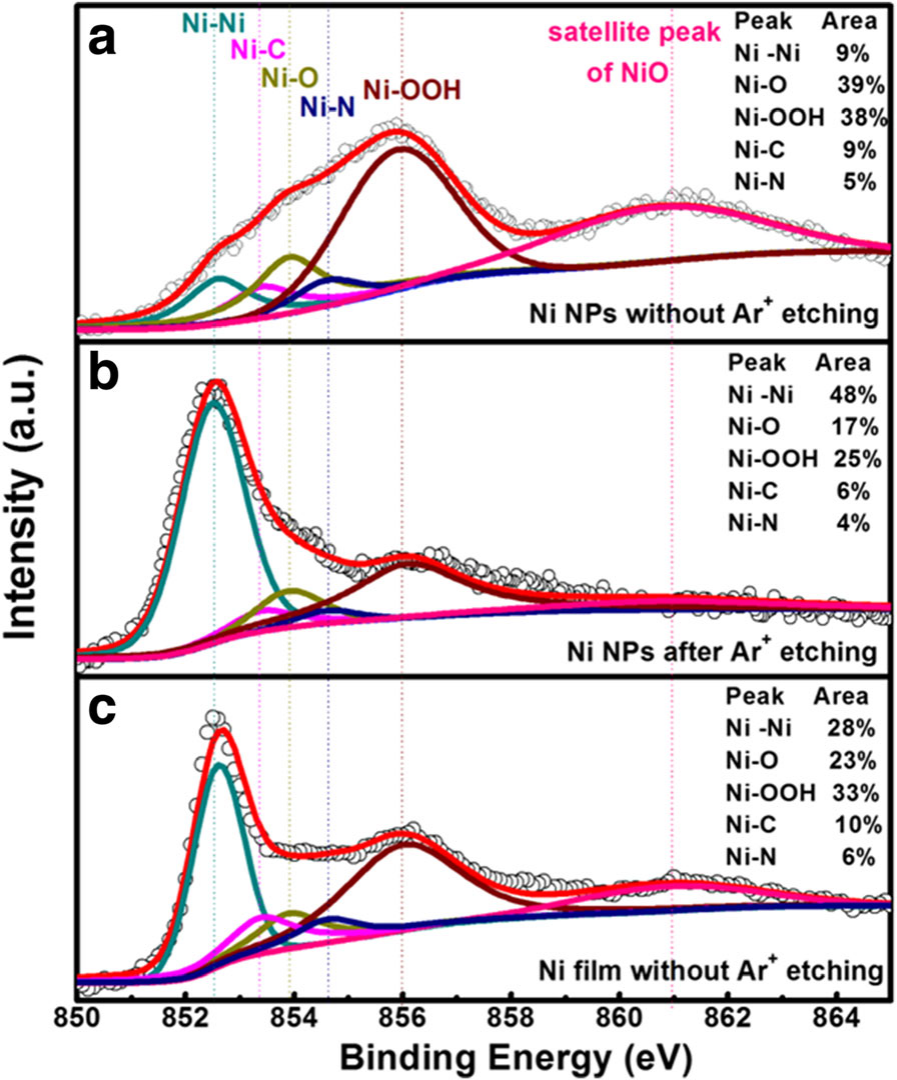
High-resolution Ni 2p_3/2_ XPS spectra of ALD Ni NPs deposited at 280 °C for 50 deposition cycles. **a** Before Ar ion etching. **b** After Ar ion etching. **c** High-resolution Ni 2p_3/2_ XPS spectrum of 20-nm ALD Ni film deposited at 280 °C before Ar ion etching

For the sake of finding out the chemical bonds and composition of the ALD Ni NPs, high-resolution Ni 2p_3/2_ XPS spectra of the Ni NPs deposited at 280 °C for 50 deposition cycles were collected and analyzed. Figure 5 shows high-resolution Ni 2p_3/2_ XPS spectra without Ar ion etching and those with in situ Ar ion etching. Each Ni 2p_3/2_ spectrum can be well separated into six components using Gaussian-Lorentian function. The peaks located at 852.6 ± 0.1 eV and 853.9 eV should be attributed to metallic Ni atoms (Ni^0^) [24, 25] and Ni–O bonding configuration in NiO structure [26, 27], respectively. And the peak located at 856 ± 0.1 eV corresponds to NiOOH [26, 28, 29]. In addition, the peak at the largest binding energy (860.8 ± 0.1 eV) is ascribed to a satellite peak due to a shake-up process in the NiO structure [26, 27]. Finally, the two peaks centered at 853.4 and 854.6 eV should be associated with Ni–C and Ni–N bonds, respectively [23]. In terms of the sample without Ar ion etching, the relative percentage of Ni^0^ is 9%, which is much smaller than that (48%) of the sample with Ar ion etching. Moreover, higher percentages of NiO and NiOOH in the sample without Ar ion etching indicate that some chemical reactions occurred on the surface of Ni NPs while being exposed to air containing O_2_ and H_2_O, hence leading to the formation of Ni oxides. After Ar ion etching, the reduced percentages of NiO and NiOOH should be ascribed to incomplete removal of the Ni oxides on the surface of Ni NPs. Moreover, the chemical bonds of Ni–C and Ni–N are still observed despite weaker intensities. To further verify whether these oxides (NiO and NiOOH) were generated from surface chemical reactions of Ni NPs with the air rather than with the backing Al_2_O_3_ film, a 20-nm Ni film was deposited under the same ALD conditions. Figure 5c shows the corresponding high-resolution Ni 2p_3/2_ XPS spectrum associated with no Ar ion etching. It can be also well fitted into six peaks, corresponding to Ni^0^, Ni–C, NiO, Ni–N, NiOOH, and a shake-up process in the NiO structure, respectively. After Ar ion etching, the bonding configurations of NiO and NiOOH disappear; meanwhile, the Ni–C and Ni–N bonds still exist, as reported in our previous paper [23]. These results argue that the ALD Ni NPs consist of metallic Ni atoms and small quantities of Ni–N and Ni–C bonds, and the oxides come from surface reactions with the air. Our previous study indicates that the incorporation of Ni–N and Ni–C bonds into the Ni film can reduce the work function of the grown Ni film to a certain degree [23]. Therefore, when the ALD Ni NPs are chosen as the charge storage medium in the a-IGZO TFT memory, the impurities (e.g., Ni–C, Ni–N) embedded in the Ni NPs probably reduce the work function of the NPs, thus leading to a reduced potential well for charge storage. This could influence the data retention of the memory. On the other hand, the Ni-NiO_*x*_ core-shell NPs may provide an additional NiO_*x*_ interfacial barrier layer between Ni and Al_2_O_3_ [30], thus possibly lowering the programming and erasing efficiencies and enhancing the data retention.

**Fig. 6:**
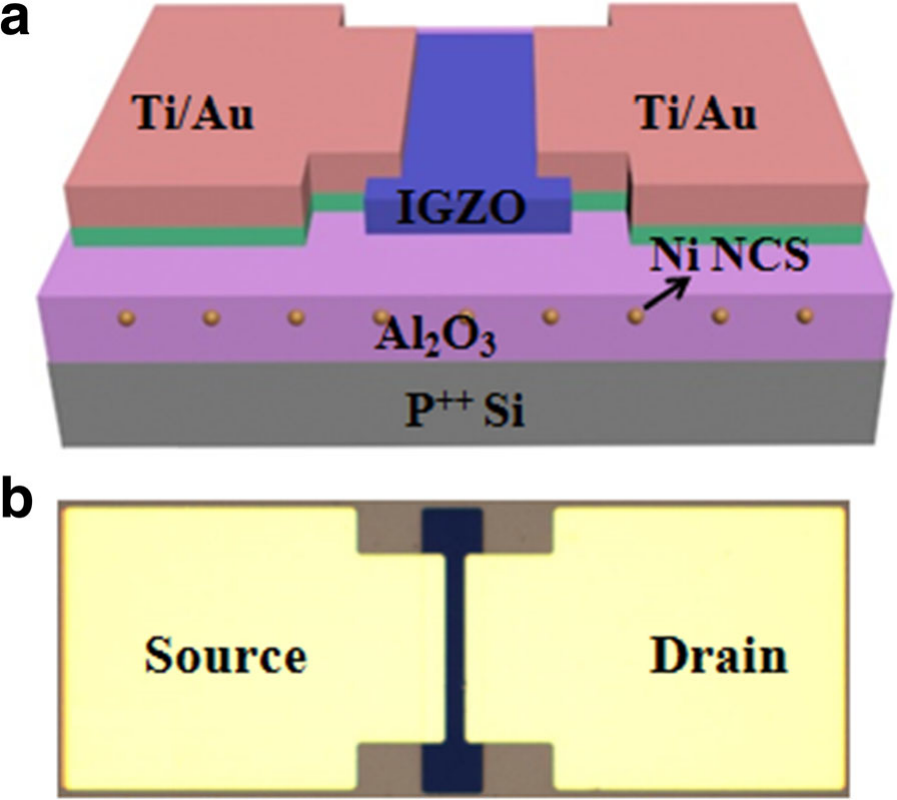
**a** Schematic diagram and **b** optical top-view image of the fabricated TFT memory

Figure 6 shows the schematic diagram and optical top-view image of the fabricated TFT memory with a Ni NP charge storage medium, which was deposited at 280 °C for 50 cycles. It is indicated that the device is a bottom-gate and top-contact TFT configuration, especially adequate overlaps between the source/drain electrode and the channel are observed, thus ensuring good contacts between them, shown in Fig. 6b.

**Fig. 7:**
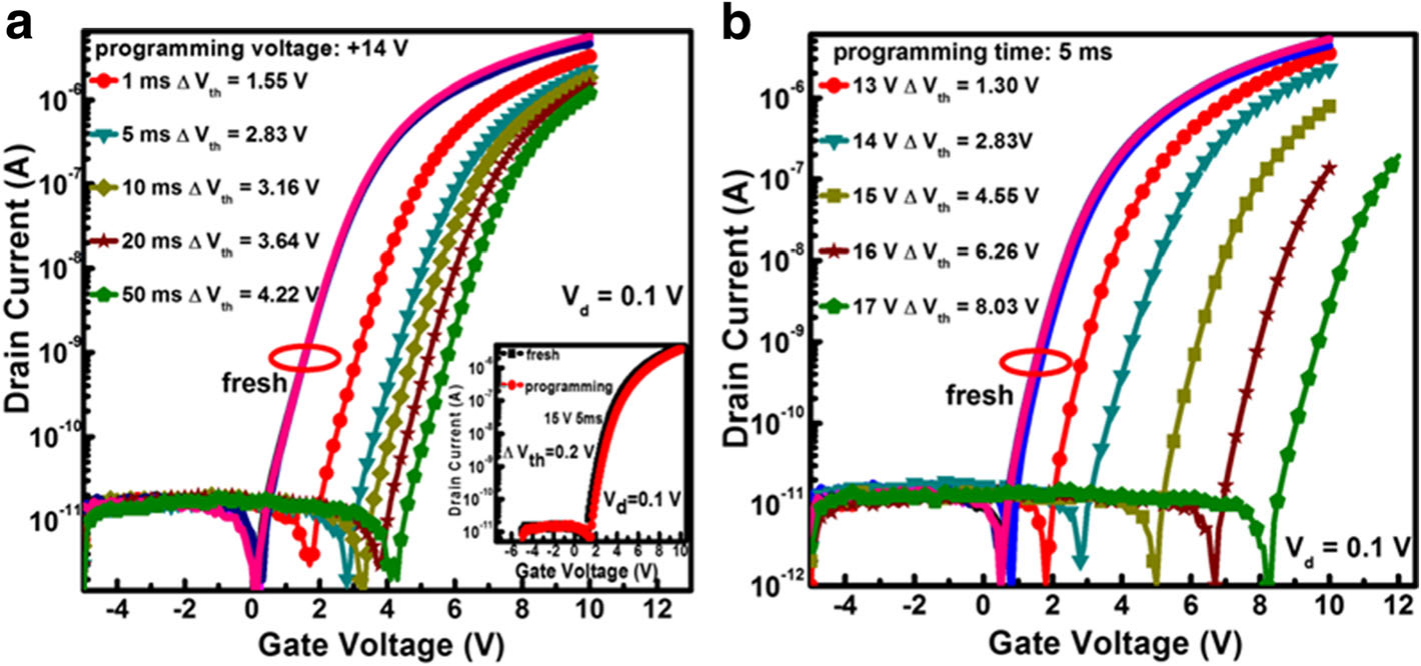
The programming characteristics of the memory. **a** Under a 14V gate bias for different programming times, the *inset* shows the programming characteristics of the control TFT without Ni NPs. **b** Under different gate biases for a constant programming time of 5 ms. During all programming operations, the source/drain electrodes were grounded

Figure 7a shows the representative programming characteristics of the memory devices for different programming times at 14 V. For each programming operation, we used a fresh device to avoid effect from the last programming cycle. It is observed that the transfer curves of all the fresh devices coincide with each other, suggesting a good electrical uniformity of the devices, denoted by the red circle. As the programming time lengthens from 1 to 50 ms, the threshold voltage shift (Δ*V*_th_) relative to the fresh device increases significantly from 1.55 to 4.22 V. However, for the control device without Ni NPs, a very small Δ*V*_th_ of 0.2 V is attained after 5 ms programming at 15 V, as shown in the inset of Fig. 7a. The results argue that the Ni NPs play a key role in electron trapping; meanwhile, the Al_2_O_3_ film has a good quality with negligible defects. Further, under a constant programming time of 5 ms, the resulting Δ*V*_th_ increases remarkably from 1.3 to 8.03 V as the programming voltage is enhanced from 13 to 17 V, as revealed in Fig. 7b.

**Fig. 8:**
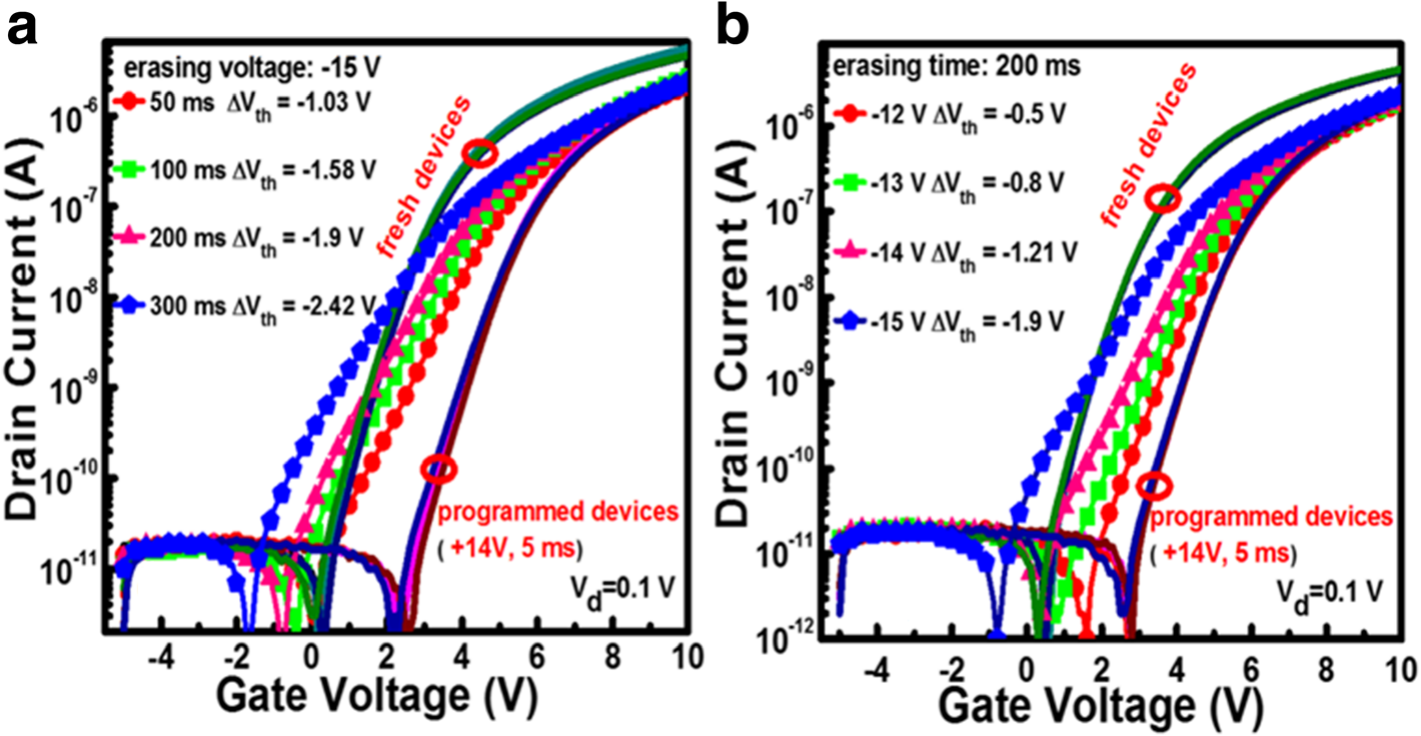
The electrical erasing characteristics of the devices programmed at 14 V for 5 ms. **a** Under a gate bias of −15 V for different times. **b** Under different gate biases for a constant erasing time of 200 ms. During all erasing operations, the source/drain electrodes were grounded

Figure 8 shows the typical electrical erasing characteristics of the programmed devices under different erasing time and voltage, respectively. When the programmed device is erased at −15 V, the resulting transfer curve shifts in the direction of negative bias as a function of erasing time. This reflects a gradual removal of the stored electrons in the NPs. That is, as the erasing time increases from 50 to 300 ms, the Δ*V*_th_ relative to the programmed device increases from −1.03 to −2.42 V, as shown in Fig. 8a. In addition, as the erasing voltage increases from −12 to −15 V, the Δ*V*_th_ increases from −0.5 to −1.9 V in the case of 200 ms erasing time, as shown in Fig. 8b. Meanwhile, the resulting transfer curves also show a deterioration trend in the sub-threshold swing (SS); this can be understood as follows. When a negative bias is applied to the gate, some neutral oxygen vacancies in the IGZO channel layer can be ionized, and these positively charged oxygen vacancies are accumulated at the IGZO/Al_2_O_3_ interface, resulting in the SS degradation [31]. Compared with the high programming efficiency of the current memory devices, the relatively low erasing efficiency can be explained as follows. Since holes can hardly be generated in the a-IGZO channel under negative gate bias because of a natural n-type semiconductor of a-IGZO [32], the programmed device can only be electrically erased by removal of electrons from the charge storage medium. However, the potential well of Al_2_O_3_/Ni NPs/Al_2_O_3_ is very deep because of the high work function of Ni; this makes the electrons trapped in the Ni NPs difficult to escape to the channel under a negative gate bias.

**Fig. 9:**
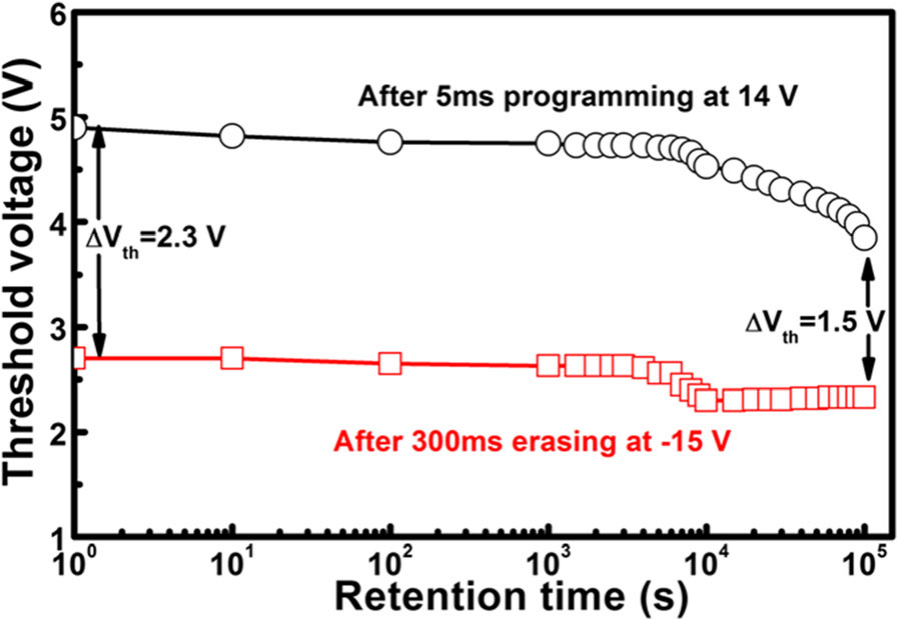
The data retention characteristics of the memory device at room temperature after 5 ms programming at 14 V and 300 ms erasing at −15 V

Figure 9 shows the data retention characteristics of the memory device after 5 ms programming at 14 V and 300 ms erasing at −15 V at room temperature. It is observed that the memory window decreases to 1.5 V with an increase in the retention time of up to 10^5^ s, retaining a memory window of 65% initial one after 10^5^ s.

## Conclusions

The growth of Ni NPs on the Al_2_O_3_ film has been investigated by plasma-assisted ALD using NiCp_2_ and NH_3_ precursors. By optimizing substrate temperature and deposition cycles, high-density (~1.5 × 10^12^ cm^−2^) and small size (3~4 nm) Ni NPs have been achieved at 280 °C in the case of 50 cycles. By using the above Ni NPs as the charge storage layer, the a-IGZO TFT memory was fabricated with the ALD gate stack of Al_2_O_3_/Ni NPs/Al_2_O_3_ assembled in the same chamber. The memory exhibits a high programming efficiency and a large programming window, which should be attributed to high-density Ni NPs. Therefore, plasma-assisted ALD Ni NPs provide a feasible approach to prepare large-area and high-density Ni NPs at relatively low temperatures for nonvolatile TFT memory applications.
